# Combining BeEAM with Brentuximab Vedotin for High-Dose Therapy in CD30 Positive Lymphomas before Autologous Transplantation—A Phase I Study

**DOI:** 10.3390/jcm11185378

**Published:** 2022-09-13

**Authors:** Christian Rausch, Ulrike Bacher, Manuela Rabaglio, Corinne Vorburger, Anke Klingenberg, Yara Banz, Michael Daskalakis, Thomas Pabst

**Affiliations:** 1 Department of Medical Oncology, Inselspital, University Hospital Bern, University of Bern, 3010 Bern, Switzerland; 2 Medical Department III—Hematology and Oncology, Campus Grosshadern, Ludwig Maximilian’s University Munich, 81377 Munich, Germany; 3 Department of Hematology, Inselspital, University Hospital Bern, University of Bern, 3010 Bern, Switzerland; 4 Institute of Pathology, University of Bern, 3008 Bern, Switzerland

**Keywords:** autologous stem cell transplantation (ASCT), CD30 lymphoma, Hodgkin, angioimmunoblastic T-cell lymphoma (AITL), brentuximab vedotin (BV)

## Abstract

The prognosis for patients with CD30+ lymphomas (Hodgkin lymphoma and various T-cell lymphomas) relapsing after autologous stem cell transplantation (ASCT) is critical. Brentuximab vedotin (BV), an ADC targeting CD30, is an obvious candidate for inclusion into high-dose chemotherapy (HDCT) regimens to improve outcomes. This single center phase I trial investigated 12 patients with CD30+ lymphoma (AITL: *n* = 5; relapsed HL: *n* = 7; median of two previous treatment lines) undergoing ASCT. In a 3 + 3 dose escalation design, 12 patients received a single BV dose at three dose levels (DL) (0.9/1.2/1.8 mg/kg b.w.) prior to standard BeEAM. All patients were treated as planned; no dose limiting toxicities (DLTs) occurred at DL 1 and 2. At DL 3, one DLT (paralytic ileus, fully recovering) occurred. Grade III febrile neutropenia occurred in one patient, and two others had septic complications, all fully recovering. Median hospitalization was 23 days. Hematologic recovery was normal. Six of twelve (50%) patients achieved CR. PFS and OS at 1 year were 67% (*n* = 8/12) and 83% (*n* = 10/12), respectively. The addition of brentuximab to standard BeEAM HDCT seems to be safe. We observed a CR rate of 75% post-ASCT in a highly pretreated population. The efficacy of this novel HDCT combination with BV at a 1.8 mg/kg dose level needs to be explored in larger studies.

## 1. Introduction

CD30+ lymphomas comprise Hodgkin lymphomas (HL), but also a variety of T-cell lymphoma types including angioimmunoblastic T-cell lymphomas (AITL), anaplastic large-cell T-cell lymphomas (ALCL), Sézary syndrome, peripheral T-cell non-Hodgkin lymphoma (T-NHL) NOS, and numerous rare malignant T-cell lymphoma entities. Standard treatment for these malignancies commonly involves high-dose chemotherapy (HDCT) with autologous stem cell transplantation (ASCT), be it in a frontline or relapse setting. In relapsed or refractory (*r/r*) HL, HDCT with ASCT cures roughly half of all patients [1]. In T-cell lymphomas, ASCT in first remission is the preferred consolidation option to improve outcomes, with one study reporting 5-y PFS of up to 49% in AITL [2]. One option as a conditioning regimen before ASCT for these entities is the BeEAM regimen (bendamustine, etoposide, cytarabine, and melphalan) as it offers comparable efficacy to the BEAM (BCNU, etoposide, cytarabine, and melphalan) regimen, avoids BCNU-related pulmonary toxicities, and its commercial availability is decisively more reliable [3,4,5,6,7,8]. However, various other drug combinations have been or are being evaluated [9,10].

Brentuximab vedotin (BV) is an antibody drug conjugate combining an anti-CD30 antibody with monomethyl auristatin E, an antimitotic cytostatic agent.

In *r*/*r* HL, BV has been studied as a single agent, showing promising response rates of around 45%, with it potentially being curative in a minority of patients with long-lasting remissions [11,12,13,14]. BV improved median PFS by about 18 months when used as a monotherapy for maintenance treatment after ASCT in *r*/*r* HL [15]. Additionally, BV together with different chemotherapies (bendamustine, DHAP, ESHAP) or checkpoint-inhibitors (ipilimumab, nivolumab) has shown promising efficacy in the salvage therapy of *r*/*r* cHL [16,17,18,19,20].

In relapsed ALCL, BV monotherapy induced CR in 57% of patients in a phase II trial [21]. In CD30+ PTCL first-line treatment, a randomized phase 3 trial showed a survival advantage of CHP (cyclophosphamide, doxorubicin prednisone) and BV over CHOP [22,23]. These data led to the approval of BV for the use in *r*/*r* HL after the failure of two prior therapy lines and as a maintenance treatment after ASCT, as well as for monotherapy in the treatment of *r*/*r* sALCL. Thus far, BV has not been studied as part of an HDCT regimen before ASCT in CD30+ lymphomas or other malignancies. In this study, therefore, we studied the safety and feasibility of the combined use of BV together with the BeEAM regimen before ASCT in CD30+ lymphoma types. Since response rates decline with every further line of therapy and the prognosis for those relapsing after ASCT is especially poor, reaching a deep and durable response with ASCT is paramount. BV is a perfect candidate for the induction of even deeper remission. BV can conceivably be added to an already quite intense therapeutic regimen due to its favorable toxicity profile. It also offers an additional targeted mechanism of action against CD30+ cells. This study therefore evaluates the feasibility of adding a relatively tolerable targeted agent to ASCT to improve the depth of response and subsequent clinical outcomes.

## 2. Patients and Methods

### 2.1. Study Design

This phase I trial is a single-arm, single-center, non-randomized open-label trial at the University Hospital in Bern, Switzerland. All patients gave written informed consent. The study protocol and related amendments were approved by the appropriate ethics committee, with the initial decision number of the ethics committee Bern, Switzerland #2016-01351 and the approval date of 24 August 2017. The study was conducted in accordance with the declaration of Helsinki (2013) and the International Conference on Harmonization Guideline for Good Clinical Practice, and it is registered as EudraCT (#2015-004266-28) and NCT03187210.

### 2.2. Inclusion and Exclusion Criteria

Key inclusion criteria included the diagnosis of CD30 positive T-cell lymphoma or HL in first or second remission or a second chemo-sensitive relapse in patients considered fit to undergo subsequent HDCT/ASCT. Prior use of BV was permitted. Diagnosis of the local pathologists was confirmed by central reference pathology (Y.B.). Further inclusion criteria comprised age of 18 to 65 years, serum creatinine < 2.0 mg/dL, transaminases of less than 3× the upper reference range, and hemoglobin level ≥ 80 g/L. Key exclusion criteria were concurrent malignant disease (except non-melanoma skin cancer and early stage cervical or prostate cancer), uncontrolled cardiovascular or infectious disease, pregnancy or lactation. We list all inclusion and exclusion criteria in the Supplementary Table S1.

### 2.3. Study Intervention

All patients were treated with standard BeEAM high-dose chemotherapy (HDCT) supported by subsequent autologous stem cell transplantation (ASCT). The regimen consisted of bendamustine 200 mg/m^2^/day given as a single infusion in 500 mL NaCl 0.9% over 120 min on days −7 and −6 and supported by forced hydration with 2000 mL NaCl 0.9%. Etoposide 200 mg/m^2^/day and cytarabine 400 mg/m^2^/day were given as a single infusion in 500 mL NaCl 0.9% over 30 min on days −5 through −2. Melphalan 140 mg/m^2^, given as a single infusion in 500 mL NaCl 0.9% over 60 min, was given on day − 1, also supported with forced hydration with 2000 mL NaCl 0.9%.

A minimum of 2.0 × 10^6^ autologous stem cells/kg body weight (b.w.) were given for ASCT at day 0. In addition to the standard BeEAM regimen, patients received three increasing dose levels of BV at day −8 according to a 3 + 3 dose escalation design. Depending on the dose level (DL), the BV dose was 0.9 mg/kg b.w. (DL 1), 1.2 mg/kg (DL 2), or 1.8 mg/kg (DL 3), suspended in sterile water (5 mg/mL) and given as a single intravenous infusion over 30 min.

We administered antiemetic prophylaxis according to local standards. All patients received valaciclovir (500 mg twice daily) for three months after ASCT, and trimethoprim/sulfamethoxazole (160 mg/800 mg three days a week) for three weeks after ASCT, as well as fluconazole (400 mg once weekly) until three weeks after ASCT. Patients received platelet or red blood cell transfusions when platelets dropped below 10 × 10^9^/L or hemoglobin below 80 g/L.

Per dose level (DL), a cohort of three patients was enrolled. If no DLT occurred, a subsequent cohort of three patients was treated at the next DL. Otherwise, another cohort of three patients at the same DL (one DLT) or at the next lower DL (>1 DLT) was treated. If no DLT occurred after treating three patients at all three DLs, another cohort of three patients at this particular maximum DL was treated to confirm tolerability at this dose.

Patients were hospitalized for the entire HDCT procedure, starting with the application of BV, and discharged after adequate physical and hematologic recovery. BV was provided free of charge while commercial medication was used for the other components of the HDCT regimen. All patients with cHL were planned to undergo BV maintenance after ASCT.

### 2.4. Study Assessments and Definitions

Patients were observed for a period of 12 months after ASCT, and after that on a yearly basis or until study discontinuation. Causes for discontinuation were patient preference, lack of compliance, relevant protocol violation, unacceptable toxicity, or death. The data cut-off for this study was 1 August 2021.

Staging was performed according to the Ann Arbor criteria and the International Prognostic Index (IPI). Standard histopathology, immunohistochemistry, and molecular studies were performed. During the entire duration of the study, all adverse events (AEs) and serious adverse events (SAEs) were collected, fully investigated, and documented, and SAEs and AEs were assessed using the NCI CTCAE v4.03.

DLT included any trial-related death or non-hematologic AEs grade ≥ III occurring between day − 8 and day + 30 that were possibly, probably, or definitely related to BV (with the exception of clinically non-relevant non-hematological laboratory findings). Hematologic DLT comprised failure to recover to an absolute neutrophil count (ANC) of >0.5 × 10^9^/L and a platelet count >20 × 10^9^/L at day + 30 without platelet substitution in the three preceding days. Finally, one or more missed therapy day(s) of BeEAM chemotherapy due to trial drug-related toxicities was also considered as a DLT.

The primary endpoint of this trial was the occurrence of an investigator-assessed DLT between day − 8 and day + 30. SAEs occurring until d + 100 and AEs occurring until d + 30 were secondary endpoints. Further secondary safety endpoints were engraftment and time to hematologic recovery. Secondary efficacy endpoints were overall survival (OS), progression free survival (PFS), remission status at day + 100, and duration of response. Time to event was calculated starting at ASCT.

Response assessment was performed at day + 100 and every 12 weeks afterwards until tumor progression or completion of the study. PET-CT was used preferentially and CT was used if PET-CT was unavailable, Response assessment was performed by the investigators in accordance with the International Harmonization Project Group 2007 Revised Response Criteria [24].

### 2.5. Statistical Analyses

The dose escalation cohorts were defined using the Fibonacci increment rule. Summary statistics presented for quantitative variables are summarized using medians and range. The summary statistics presented for categorical data are presented as the absolute frequency and percentage of patients in each category. AEs are presented by type and grade using frequency and percentage of the within-patient worst grades. In addition, grade ≥ 3 AEs and AEs related to trial treatment (relation to trial treatment “possible”, “probable”, or “definite”) are summarized separately.

For secondary efficacy endpoints expressed as a rate, the point estimate of the rate and the associated 95% CI were calculated. All time to event endpoints had the median value estimated using the Kaplan–Meier method, along with a 95% CI. The types of events of each endpoint were presented descriptively by frequency and percentage. Early and late toxicities were compared using Fisher’s exact test. All calculations were performed on a strict intention-to-treat basis. *p*-values < 0.05 were considered significant. Data were analyzed using Graphpad Prism^®^ Version 9.4.0 (Graphpad Software Inc., La Jolla, CA, USA).

## 3. Results

### 3.1. Patient Characteristics

We studied 12 patients with CD30+ lymphomas treated at a single center between 09/2018 and 09/2020. Seven patients had classical Hodgkin lymphoma (HL), and five patients had angioimmunoblastic T-cell lymphomas (AITL). All AITL were transplanted as consolidation in first remission, while HLs were transplanted as salvage therapy in first (*n* = 5) or second (*n* = 2) relapse. The median age at diagnosis was 56 years (range, 19 to 63 years), with the expected male predominance (8/12 patients), and a median interval from diagnosis to HDCT/ASCT of 8 months (range 6 to 148 months). At ASCT, eight patients were in CR and four were in PR. Before HDCT/ASCT, patients had received between one and three prior lines of therapy (median, two lines). Four patients had been exposed to BV in prior lines of therapy, but were not refractory or intolerant to BV. Four patients with cHL received BV maintenance after ASCT. The median follow-up was 23 months. Patient characteristics are summarized in Table 1.

### 3.2. Treatment

All patients received the complete HDCT following the BV-BeEAM protocol as planned and without any dose reduction. The median CD34+ number administered was 5.6 × 10^6^ CD34+ cells/kg (range 2.8–11.6). G-CSF was administered subcutaneously from d + 6 until d + 12 after ASCT at a daily dose of 30 Mio U/d (for patients < 78 kg) or 48 Mio U/d (for patients ≥78 kg). Patients received a median of three units of red blood cells (range, 0–9) and four units of platelets (range 2–8) during hospitalization.

### 3.3. Hematologic Recovery and Hospitalization Duration

All patients at all three dose levels experienced hematologic recovery within 30 days after ASCT. The median interval from ASCT to neutrophil recovery (ANC > 1.0 × 10^9^/L) was 12 days (range 10 to 25 days). The median interval to platelet recovery (Tc > 20 × 10^9^/L) was 15 days (range 10 to 28 days). Details on hematologic recovery and toxicities are shown in Table 2. A systematic comparison of engraftment times to previously published studies using BeEAM HDCT before ASCT is shown in Supplementary Table S2. Finally, the median hospitalization duration was 23 days, ranging from 22 to 56 days.

### 3.4. Non-Hematologic Toxicities

Non-hematologic toxicities as defined in the protocol were as follows. During hospitalization, relevant oral mucositis (grade 3) occurred in one patient at DL2, for which an association to BV was considered unlikely. Another patient, who was treated at the highest dose level (BV 1.8 mg/kg b.w.), developed steroid-induced diabetes mellitus and paralytic ileus (both grade 3) while hospitalized. Since paralytic ileus due to autonomic neuropathy has been described in 7.7% of patients in one retrospective study [25], and since there was no other obvious explanation such as intestinal manifestation of the lymphoma, we considered the ileus potentially related to treatment with BV. This AE was therefore classified as a DLT. Fortunately, within eight days the ileus completely resolved under supportive therapy.

While uncontrolled diabetes mellitus after BV application has been reported in three case reports, we still consider a relation to BV highly unlikely in our case. Two patients had to be treated in the ICU and no treatment-related deaths occurred. These toxicities were considered as expected to occur during BeEAM HDCT and/or its comedication. A systematic comparison of toxicities in this cohort compared to other published cohorts using BeEAM HDCT is shown in Supplementary Table S3. Late non-infectious toxicities were not observed.

### 3.5. Infectious Complications

All patients had at least one febrile episode (≥38.0 °C) while being hospitalized. Febrile episodes grade 1 with no identified infectious focus were the most common infectious complication (*n* = 20). One grade III febrile neutropenia occurred in a patient treated at dose level 2. Two patients who received the maximum dose of BV had septic complications (grade 4), requiring transient ICU admission with complete recovery in both patients. Five patients developed lower airway infections needing antibiotic treatment between discharge from the hospital after the HDCT treatment and the day + 100 evaluation. Two were treated as outpatients (grade 2, one each at DL 1 and 2) while three required re-hospitalization (grade 3, two at DL2 and one at DL3). No CMV reactivation was observed in the 100 days after ASCT. Again, a relationship to the study drug (BV) administration was considered unlikely for these infectious complications. Non-hematologic toxicities and infectious complications are summarized in Table 2.

### 3.6. Outcomes

**Figure 1:**
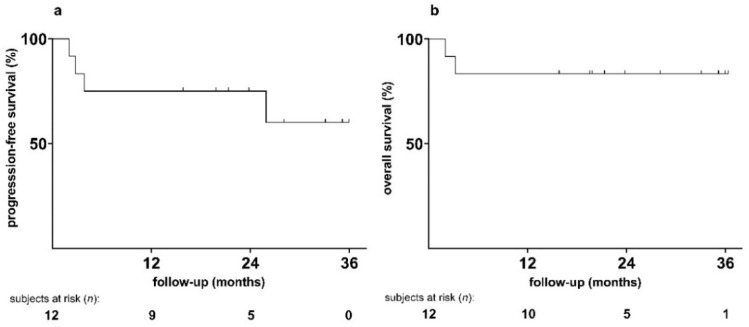
Kaplan–Meier estimator of cumulative survival rates of 12 patients with CD30 positive lymphoma after HDCT with BV-BeEAM followed by ASCT: (**a**) progression free survival; (**b**) overall survival.

The outcomes of the study cohort are summarized in Table 3 and Figure 1. Supplementary Figure S1 shows EFS and OS by histopathology. After a median follow-up of 23 months, the PFS rate was 8/12 (67%) and the OS was 10/12 (83%) patients. No patient was lost to follow-up. Neither the median PFS nor the median OS were reached.

Before ASCT, seven patients were in CR, and none of them progressed until the day + 100 evaluation. Thus, the remissions status of these seven patients remained CR. Among the remaining five patients not in CR at HDCT/ASCT, two patients achieved CR at day + 100, whereas three patients were progressive, with two deaths among them due to lymphoma progression before day + 100 (both AITL). At the follow-up after one year, one patient had relapsed and all other CRs were ongoing. Thus, the CR rate was 8 out of 12 patients (67%) at the last follow-up. Interestingly, no patient who had been exposed to BV in prior lines of therapy relapsed. (Supplementary Figure S2).

## 4. Discussion

This study investigated the use of BV in combination with HDCT before ASCT in the treatment of CD30 positive lymphomas. While BV is currently used as a maintenance therapy in relapsed HL after ASCT, response rates in CD30+ lymphomas decrease with every further line of therapy [15,21,26]. Especially for patients relapsing after ASCT, the outcome is poor, suggesting an urgent need for improving outcomes following HDCT/ASCT [27,28]. Therefore, achieving high rates of durable remissions following HDCT/ASCT is crucial. In this study, we evaluated whether the combination of standard HDCT (BeEAM) with BV is safe and whether we could observe signals for added efficacy in patients with CD30 positive lymphomas.

Previously, Sirohi et al. reported a 10-year OS of 72%, 54%, and 11%, respectively, for patients with *r/r* HL reaching CR, PR, or no response prior to ASCT, using different salvage chemotherapy regimens (ABVD, BVAP/EVAP, ClhVPP, EPIC, GEM-P, Hope-Bleo, HYBRID, PmitCEBOM, VEEP, among others) [29]. In another study in *r/r* cHL patients, Moskowitz et al. documented a 10-year OS difference of 66% vs. 17% when comparing responders vs. non-responders to second-line chemotherapy prior to ASCT. [30] The same has been shown for PTCL, with a significantly longer 3-year OS (80% vs. 65%) after ASCT achieved in chemo-sensitive patients [31]. These results identify the response to salvage therapy as a key predictor of survival after ASCT in CD30 positive lymphomas. In this study, we therefore combined BV with standard HDCT to explore the safety and efficacy of a novel HDCT regimen (BV-BeEAM).

First, the novel combination of BV with BeEAM appeared safe in our study. The median duration of hospitalization was 23 days with BV-BeEAM, which is similar to the 27 days observed by our group and the 24 days observed in two studies conducted by a Canadian and a Dutch group using BeEAM alone in patients with various NHL and HL [3,4,6]. These studies reported a median neutrophil engraftment of 10–12 days and platelet engraftment of 13–15 days for BeEAM alone. In this study, we documented a median neutrophil engraftment of 11 days and a median platelet engraftment of 15 days, which is well in range of the above studies with BeEAM HDCT. The ICU admission rate in our study was 16%, which also compared favorably to our previous results with BeEAM alone published by Gilli et al., reporting an ICU admission rate of 10% [3]. We observed no treatment-related deaths in our cohort. Infectious complications represented the most frequent AE occurring in all patients after the BV-BeEAM regimen. At least one pathogen was identified in 58% (7/12) of patients. This was similar to our previous experiences in lymphoma patients receiving BeEAM alone with a pathogen identification rate of 79% [3]. Grade 1 febrile episodes were frequent across all dose levels (7 at DL 1, 8 at DL 2, 5 at DL 3), while more serious infectious complications were more frequent at higher dose levels, with no infectious complications of grade 3 at DL 1 and no grade 4 infectious AEs at DL 1 and DL 2. Due to the small number of patients and the expectation of grade 3 and 4 infectious AEs with BeEAM alone, we maintain however, that a relation to BV should still be considered unlikely.

AT DL 3, one case of grade 3 therapy-associated diabetes occurred. Three case reports describe an association of BV and diabetes. In one report, insulin resistance occurred in the context of a rapidly lethal cytokine release syndrome, which did not occur in our patient [32]. In two cases, DM occurred at least ten days after the application of BV or corticosteroids and did not resolve after the discontinuation of treatment [33,34]. Conversely, glucose levels of our patient were elevated during HDCT, for which the patient received dexamethasone as antiemetic comedication, and returned to normal levels shortly after the discontinuation of dexamethasone. Therefore, we believe, that the diabetes in our patient was steroid-induced, and not related to BV.

Furthermore, we focused on the question of pulmonary toxicity that may be linked to BV. In the treatment arm of the AETHERA trial, which evaluated BV as early consolidation therapy after ASCT in *r/r* HL, 5% of patients had treatment-related pulmonary toxicities, and two related deaths occurred [15]. Interestingly, in the present study, we observed no pulmonary toxicities. We observed no relevant peripheral neuropathy in our population, in contrast to the experiences of the AETHERA trial with a PNP rate of 56%. However, the occurrence of a paralytic ileus at DL 3 raises the potential of autonomic neuropathy in one patient treated at DL 3. One retrospective study reported paralytic ilei subsequent to autonomic neuropathy in 7.7% of patients following BV [25]. Since there was no other obvious explanation for the ileus in our patient (e.g., intestinal lymphoma involvement), we also consider this the likely explanation in our case. Fortunately, using only supportive therapy, the ileus resolved completely within 8 days.

Regarding the efficacy of the new regimen, our study suggested a CR rate of 75% after BV-BeEAM/ASCT. The overall response rate of this phase I study appears promising, as larger studies in this setting reported a CR rate of 72% and 74%, respectively, using BeEAM in *r/r* lymphoma patients [3,35]. PFS at one year was 67% in our study, while the aforementioned studies reported a 75% one-year PFS and 81% eighteen-month DFS respectively. OS data show a similar picture, with an 83% one-year OS rate in our study and 72% two-year OS and 95% one-year OS in the other studies. However, these cohorts from the literature did not contain any T-cell lymphomas, which carry a markedly poorer prognosis. Additionally, as most patients in our study received BV at a lower dose than that recommended for a phase II study, results of a phase II study might be even more beneficial.

Taking the above aspects together, BV-BeEAM before ASCT was safe and well tolerated, no unexpected toxicities occurred and toxicities were largely as expected for BeEAM alone. We applied BV at all three preplanned dose levels without the emergence of unexpected toxicities. No DLTs occurred at dose levels one and two, and one DLT occurred in the second group of three patients treated at DL 3 (paralytic ileus). We have therefore successfully identified the highest dose level of BV (1.8 mg/kg BW), which is equal to approved indications, as the recommended dose level for a phase 2 dose expansion study. Due to the small number of subjects and the short follow-up, the efficacy of the novel BV-BeEAM regimen remains to be further studied. Nonetheless, with a CR rate of 75% as well as an OS rate and PFS rate at one year of 83% and 67%, respectively, in a patient group that usually has an adverse prognosis, the novel approach warrants further evaluation as an HDCT regimen before ASCT in patients with CD30 positive lymphomas.

## Figures and Tables

**Table 1:** Patient characteristics.

Demographic Characteristics	Results, All	Results, AITL ^2^	Results, cHL ^3^
Patients, *n*	12	5 (42%)	7 (58%)
Age at diagnosis, y, median (range)	56 (19–63)	58 (55–63)	25 (19–62)
Age at ASCT ^1^, y, median (range)	56 (19–64)	58 (55–64)	25 (19–62)
Male/female ratio	3:1	2:3	7:0
**Histopathology**			
CD30+ lymphocytes (histopathology at diagnosis), %, median (range)	85 (1–100)	5 (1–10)	95 (80–100)
**Stage at diagnosis (Ann Arbor), *n* (%)**			
I	1 (8%)	0 (0%)	1 (14%)
II	2 (17%)	0 (0%)	2 (29%)
III	4 (33%)	2 (40%)	2 (29%)
IV	5 (42%)	3 (60%)	2 (29%)
**International Prognostic Index (IPI) at diagnosis, *n* (%)**			
0		0 (0%)	NA
1		0 (0%)	NA
2		2 (40%)	NA
3		3 (60%)	NA
**Clinical manifestations at diagnosis, *n* (%)**			
B-symptoms	8 (67%)	4 (80%)	4 (57%)
Extranodal involvement	5 (42%)	2 (40%)	3 (43%)
Bone marrow infiltration	0 (0%)	0 (0%)	0 (0%)
Spleen	2 (17%)	2 (40%)	0 (0%)
Mediastinum	2 (17%)	0 (0%)	2 (29%)
Other	3 (25%)	2 (40%)	1 (14%)
**Time from Diagnosis to ASCT ^1^, m, median (range)**	8 (5–148)	6 (5–7)	25 (7–148)
**Previous therapies**			
Prior lines of chemotherapy, median (range)	2 (1–3)	1 (1–2)	3 (2–3)
**First-line therapies**			
ABVD ^5^, *n* (%)	3 (25%)	0 (0%)	3 (43%) ^3^
BEACOPP ^6^, *n* (%)	2 (17%)	0 (0%)	2 (29%)
CHOP ^7^, *n* (%)	3 (25%)	3 (60%)	0 (0%)
Others, *n* (%)	4 (33%)	2 ^9^ (40%)	2 ^10^ (29%)
**Subsequent therapies**			
BEACOPP ^6^, *n* (%)	1 (8%)	0 (0%)	1 (14%)
DHAP ^4^, *n* (%)	4 (33%)	0 (0%)	4 (57%)
BV ^8^, *n* (%)	1 (8%)	0 (0%)	1 (14%)
Radiotherapy, *n* (%)	2 (17%)	0 (0%)	2 (29%)
Others ^9^, *n* (%)	5 (42%)	1 ^11^ (20%)	4 ^12^ (57%)
**Remission status before ASCT ^1^, *n* (%)**			
Complete remission (CR)	8 (67%)	3 (60%)	5 (71%)
Partial remission (PR)	4 (33%)	2 (40%)	2 (29%)

^1^ Autologous stem cell transplant; ^2^ angioimmunoblastic T-cell lymphoma; ^3^ classical Hodgkin’s lymphoma; ^4^ dexamethasone, high-dose cytarabine, and cisplatin; ^5^ doxorubicin, bleomycin, vinblastine, and dacarbazine; ^6^ bleomycin, etoposide, doxorubicin, cyclophosphamide, vincristine, procarbazine, and prednisone; ^7^ cyclophosphamide, doxorubicin, vincristine, and prednisone; ^8^ brentuximab vedotin; ^9^ A + CHP (brentuximab vedotin, cyclophosphamide, doxorubicin, and prednisone, *n* = 1), CHOEP (cyclophosphamide, doxorubicin, etoposide, vincristine, and prednisone *n* = 1); ^10^ BrECADD (brentuximab vedotin, etoposide, cyclophosphamide, adriamycin, dacarbazin, and dexamethasone *n* = 1), Stanford V (mechlorethamine, doxorubicin, vinblastine, vincristine, bleomycin, etoposide, and prednisone, *n* = 1); ^11^ A + CHP (brentuximab vedotin, cyclophosphamide, doxorubicin, and prednisone, *n* = 1); ^12^ DHAO (dexamethasone, high-dose cytarabine, and oxaliplatin *n* = 2), ESHAP (etoposide, methylprednisolone, high-dose cytarabine, and cisplatin, *n* = 1), IGEV (ifosfamide, gemcitabine, vinorelbine, and prednisone, *n* = 1).

**Table 2:** Engraftment and toxicities at all dose levels.

Application of HDCT/ASCT	Results
BeEAM + BV ^1^ protocols given as planned, *n* (%)	12 (100%)
Transplanted CD34+ cells, ×10^6^/kg b.w., median (range)	5.6 (2.8–11.5)
**Interval to hematological recovery, days, median (range)**	
Tc ^2^ > 20 × 10^9^/L	15 (10–28)
Tc > 50 × 10^9^/L	26 (16–86)
Tc > 100 × 10^9^/L	40 (25–86)
ANC ^3^ > 0.5 × 10^9^/L	11 (8–17)
ANC > 1.0 × 10^9^/L	12 (10–25)
**Peripheral blood product use, median (range)**	
RBC units ^4^	3 (0–9)
Tc units ^2^	4 (2–8)
**Duration of Hospitalization, d, median (range)**	**23 (22–56)**
**Adverse events**	
Patients with at least one AE ^5^ (all grades), *n* (%)	12 (100%)
Patients with at least one AE (grade III–IV), *n* (%)	4 (33%)
Patients with more than one AE (all grades), *n* (%)	7 (58%)
Severity of AEs, graded by CTCAE ^6^, median (range)	1 (1–4)
AEs ^5^ possibly, probably, or definitely related to trial treatment, *n* (%)	1 (3%)
**Adverse events by type, *n* (%)**	
Mucosal ^7^	1 (3%)
Infectious ^8^	27 (90%)
Other ^9^	2(7%)
**Infections**	
Patients with at least one febrile episode (>38.0 °C), *n* (%)	12 (100%)
Febrile episodes (>38.0 °C), median (range)	2 (1–4)
Patients with at least 1 identified pathogen, *n* (%)	7 (58%)
Patients with >1 identified pathogen, *n* (%)	1 (8%)
Bacteria, Gram-positive ^10,11^, *n* (%)	5 (42%)
Bacteria, Gram-negative ^10,12^, *n* (%)	3 (25%)
Viral, *n* (%)	0 (0%)
Fungal, *n* (%)	0 (0%)

^1^ Brentuximab vedotin; ^2^ thrombocytes; ^3^ absolute neutrophil count; ^4^ red blood cells; ^5^ adverse event; ^6^ common terminology criteria for adverse events; ^7^ oral mucositis (*n* = 1, grade III); ^8^ febrile neutropenia (*n* = 1, grade III); pneumonia (*n* = 2, grade II and 2, grade III); sepsis (*n* = 2, grade IV); ^9^ steroid-induced diabetes mellitus (*n* = 1, grade III); paralytic ileus (*n* = 1, Grade III); ^10^ identified using cultures of blood (*n* = 6), urine (*n* = 1), and a CVK tip (*n* = 1); ^11^ coagulase negative *Streptococci* (3), *Corynebacterium* (1); ^12^ *E. cloacae* (1), *E. coli* (2).

**Table 3:** Outcomes.

	All	AITL ^1^	cHL ^2^
**Median Follow-up, months**	23	12	24
Time since ASCT, d, median (range)	687 (24–1105)	350 (24–1093)	725 (594–1105)
OS ^3^ at 1 y, *n* (%)	10 (83%)	3 (60%)	7 (100%)
PFS ^4^ at 1 y, *n* (%)	8 (67%)	3 (60%)	5 (71%)
**Remission status at d +100, *n* (%)**			
CR ^5^	10 (83%)	3 (60%)	7 (100%)
PR ^6^	0 (0%)	0 (0%)	0 (0%)
SD ^7^	0 (0%)	0 (0%)	0 (0%)
PD ^8^	2 (18%)	2 (40%)	0 (0%)
**Death, *n* (%)**			
Due to TRM ^9^	0 (0%)	0 (0%)	0 (0%)
Due to progression	2 (18%)	2 (40%)	0 (0%)
Due to other causes	0 (0%)	0 (0%)	0 (0%)

^1^ Angioimmunoblastic T-cell lymphoma; ^2^ classical Hodgkin lymphoma; ^3^ overall survival; ^4^ progression free survival; ^5^ complete response; ^6^ partial response; ^7^ stable disease; ^8^ progressive disease; ^9^ treatment-related mortality.

## Data Availability

Data are available upon request to the corresponding author.

## Supplementary Materials

The following supporting information can be downloaded at: https://www.mdpi.com/article/10.3390/jcm11185378/s1, Table S1: Eligibility criteria; Table S2: Engraftment times of patients undergoing BeEAM compared to BV-BeEAM; Table S3: Toxicities of BeEAM compared to BV-BeEAM. Figure S1: PFS and OS by entity; Figure S2: PFS and OS by prior exposure to BV.

## References

[1] Schmitz N., Pfistner B., Sextro M., Sieber M., Carella A.M., Haenel M., Boissevain F., Zschaber R., Müller P., Kirchner H. (2002). Aggressive conventional chemotherapy compared with high-dose chemotherapy with autologous haemopoietic stem-cell transplantation for relapsed chemosensitive Hodgkin’s disease: A randomised trial. Lancet.

[2] D’Amore F., Relander T., Lauritzsen G.F., Jantunen E., Hagberg H., Anderson H., Holte H., Österborg A., Merup M., Brown P. (2012). Up-Front Autologous Stem-Cell Transplantation in Peripheral T-Cell Lymphoma: NLG-T-01. J. Clin. Oncol..

[3] Gilli S., Novak U., Taleghani B.M., Baerlocher G.M., Leibundgut K., Banz Y., Zander T., Betticher D., Egger T., Rauch D. (2017). BeEAM conditioning with bendamustine-replacing BCNU before autologous transplantation is safe and effective in lymphoma patients. Ann. Hematol..

[4] Hahn L., Lim H., Dusyk T., Sabry W., Elemary M., Stakiw J., Danyluk P., Bosch M. (2021). BeEAM conditioning regimen is a safe, efficacious and economical alternative to BEAM chemotherapy. Sci. Rep..

[5] Saleh K., Danu A., Koscielny S., Legoupil C., Pilorge S., Castilla-Llorente C., Ghez D., Lazarovici J., Michot J.-M., Khalife-Saleh N. (2018). A retrospective, matched paired analysis comparing bendamustine containing BeEAM versus BEAM conditioning regimen: Results from a single center experience. Leuk. Lymphoma.

[6] Visani G., Malerba L., Stefani P.M., Capria S., Galieni P., Gaudio F., Specchia G., Meloni G., Gherlinzoni F., Giardini C. (2011). BeEAM (bendamustine, etoposide, cytarabine, melphalan) before autologous stem cell transplantation is safe and effective for resistant/relapsed lymphoma patients. Blood.

[7] Noesslinger T., Panny M., Simanek R., Moestl M., Boehm A., Menschel E., Koller E., Keil F. (2018). High-dose Bendamustine-EAM followed by autologous stem cell rescue results in long-term remission rates in lymphoma patients, without renal toxicity. Eur. J. Haematol..

[8] Redondo A.M., Valcárcel D., González-Rodríguez A.P., Suárez-Lledó M., Bello J.L., Canales M., Gayoso J., Colorado M., Jarque I., Campo R. (2018). Bendamustine as part of conditioning of autologous stem cell transplantation in patients with aggressive lymphoma: A phase 2 study from the GELTAMO group. Br. J. Haematol..

[9] Farag S., Bacher U., Jeker B., Legros M., Rhyner G., Lüthi J.-M., Schardt J., Zander T., Daskalakis M., Mansouri B. (2022). Adding bendamustine to melphalan before ASCT improves CR rate in myeloma vs. melphalan alone: A randomized phase-2 trial. Bone Marrow Transplant..

[10] Gurevich E., Hayoz M., Aebi Y., Largiadèr C.R., Taleghani B.M., Bacher U., Pabst T. (2022). Comparison of Melphalan Combined with Treosulfan or Busulfan as High-Dose Chemotherapy before Autologous Stem Cell Transplantation in AML. Cancers.

[11] Bartlett N., Grove L.E., Kennedy D.A., Sievers E.L., Forero-Torres A. (2010). Objective responses with brentuximab vedotin (SGN-35) retreatment in CD30-positive hematologic malignancies: A case series. J. Clin. Oncol..

[12] Younes A., Bartlett N.L., Leonard J.P., Kennedy D.A., Lynch C.M., Sievers E.L., Forero-Torres A. (2010). Brentuximab Vedotin (SGN-35) for Relapsed CD30-Positive Lymphomas. N. Engl. J. Med..

[13] Younes A., Gopal A.K., Smith S.E., Ansell S.M., Rosenblatt J.D., Savage K.J., Ramchandren R., Bartlett N.L., Cheson B.D., De Vos S. (2012). Results of a Pivotal Phase II Study of Brentuximab Vedotin for Patients with Relapsed or Refractory Hodgkin’s Lymphoma. J. Clin. Oncol. Off..

[14] Chen R., Gopal A.K., Smith S.E., Ansell S.M., Rosenblatt J.D., Savage K.J., Connors J.M., Engert A., Larsen E.K., Huebner D. (2016). Five-year survival and durability results of brentuximab vedotin in patients with relapsed or refractory Hodgkin lymphoma. Blood.

[15] Moskowitz C.H., Nademanee A., Masszi T., Agura E., Holowiecki J., Abidi M.H., Chen A.I., Stiff P., Gianni A.M., Carella A. (2015). Brentuximab vedotin as consolidation therapy after autologous stem-cell transplantation in patients with Hodgkin’s lymphoma at risk of relapse or progression (AETHERA): A randomised, double-blind, placebo-controlled, phase 3 trial. Lancet.

[16] Diefenbach C.S., Hong F., Ambinder R.F., Cohen J.B., Robertson M.J., David K.A., Advani R.H., Fenske T.S., Barta S.K., Palmisiano N.D. (2020). Ipilimumab, nivolumab, and brentuximab vedotin combination therapies in patients with relapsed or refractory Hodgkin lymphoma: Phase 1 results of an open-label, multicentre, phase 1/2 trial. Lancet Haematol..

[17] Garcia-Sanz R., Sureda A., Gonzalez A.P., De La Cruz F., Sanchez-Gonzalez B., Rodriguez A., Domingo-Domenech E., Miriam M., Lopez S.J., Jose P.L. (2016). Brentuximab Vedotin Plus ESHAP (BRESHAP) Is a Highly Effective Combination for Inducing Remission in Refractory and Relapsed Hodgkin Lymphoma Patients Prior to Autologous Stem Cell Transplant: A Trial of the Spanish Group of Lymphoma and Bone Marrow Transplantation (GELTAMO). Blood.

[18] Hagenbeek A., Mooij H., Zijlstra J., Lugtenburg P., Van Imhoff G., Nijland M., Tonino S., Hutchings M., Spiering M., Liu R. (2019). Phase I dose-escalation study of brentuximab-vedotin combined with dexamethasone, high-dose cytarabine and cisplatin, as salvage treatment in relapsed/refractory classical Hodgkin lymphoma: The HOVON/LLPC Transplant BRaVE study. Haematologica.

[19] O’Connor P.O.A., Lue J.K., Sawas A., Amengual J.E., Deng C., Kalac M., Falchi L., Marchi E., Turenne I., Lichtenstein R. (2018). Brentuximab vedotin plus bendamustine in relapsed or refractory Hodgkin’s lymphoma: An international, multicentre, single-arm, phase 1–2 trial. Lancet Oncol..

[20] Garcia-Sanz R., Sureda A., de la Cruz F., Canales M., Gonzalez A.P., Pinana J.L., Rodriguez A., Gutierrez A., Domingo-Domenech E., Sanchez-Gonzalez B. (2019). Brentuximab vedotin and ESHAP is highly effective as second-line therapy for Hodgkin lymphoma patients (long-term results of a trial by the Spanish GELTAMO Group). Ann. Oncol..

[21] Pro B., Advani R., Brice P., Bartlett N.L., Rosenblatt J.D., Illidge T., Matous J., Ramchandren R., Fanale M., Connors J.M. (2012). Brentuximab Vedotin (SGN-35) in Patients with Relapsed or Refractory Systemic Anaplastic Large-Cell Lymphoma: Results of a Phase II Study. J. Clin. Oncol..

[22] Horwitz S., O’Connor O.A., Pro B., Illidge T., Fanale M., Advani R., Bartlett N., Christensen J.H., Morschhauser F., Domenech E.D. (2019). Brentuximab vedotin with chemotherapy for CD30-positive peripheral T-cell lymphoma (ECHELON-2): A global, double-blind, randomised, phase 3 trial. Lancet.

[23] Horwitz S., O’Connor O., Pro B., Trümper L., Iyer S., Advani R., Bartlett N., Christensen J., Morschhauser F., Domingo-Domenech E. (2022). The ECHELON-2 Trial: 5-year results of a randomized, phase III study of brentuximab vedotin with chemotherapy for CD30-positive peripheral T-cell lymphoma. Ann. Oncol..

[24] Cheson B.D., Pfistner B., Juweid M.E., Gascoyne R.D., Specht L., Horning S.J., Coiffier B., Fisher R.I., Hagenbeek A., Zucca E. (2007). Revised Response Criteria for Malignant Lymphoma. J. Clin. Oncol..

[25] Clarivet B., Vincent L., Vergely L., Bres V., Foglia K., Cartron G., Hillaire-Buys D., Faillie J.-L. (2019). Adverse reactions related to brentuximab vedotin use: A real-life retrospective study. Therapies.

[26] Voorhees T.J., Beaven A.W. (2020). Therapeutic Updates for Relapsed and Refractory Classical Hodgkin Lymphoma. Cancers.

[27] KewalRamani T., Nimer S.D., Zelenetz A.D., Malhotra S., Qin J., Yahalom J., Moskowitz C.H. (2003). Progressive disease following autologous transplantation in patients with chemosensitive relapsed or primary refractory Hodgkin’s disease or aggressive non-Hodgkin’s lymphoma. Bone Marrow Transplant..

[28] Mak V., Hamm J., Chhanabhai M., Shenkier T., Klasa R., Sehn L.H., Villa D., Gascoyne R.D., Connors J.M., Savage K.J. (2013). Survival of Patients with Peripheral T-Cell Lymphoma After First Relapse or Progression: Spectrum of Disease and Rare Long-Term Survivors. J. Clin. Oncol..

[29] Sirohi B., Cunningham D., Powles R., Murphy F., Arkenau T., Norman A., Oates J., Wotherspoon A., Horwich A. (2008). Long-term outcome of autologous stem-cell transplantation in relapsed or refractory Hodgkin’s lymphoma. Ann. Oncol..

[30] Moskowitz C.H., KewalRamani T., Nimer S.D., Gonzalez M., Zelenetz A., Yahalom J. (2004). Effectiveness of high dose chemoradiotherapy and autologous stem cell transplantation for patients with biopsy-proven primary refractory Hodgkin’s disease. Br. J. Haematol..

[31] Rodríguez J., Conde E., Gutiérrez A., Arranz R., León A., Marín J., Bendandi M., Albo C., Caballero M.D. (2007). Frontline autologous stem cell transplantation in high-risk peripheral T-cell lymphoma: A prospective study from The Gel-Tamo Study Group. Eur. J. Haematol..

[32] Chiang J.M., Lai A.R., Anderson M., Rushakoff R.J. (2020). Severe Insulin Resistance with Diabetic Ketoacidosis After Brentuximab Treatment. AACE Clin. Case Rep..

[33] Köksalan D., Sözen M., Selek A., Gezer E., Cantürk Z., Çetinarslan B. (2022). Brentuximab vedotin-associated diabetic ketoacidosis: A case report. Int. J. Diabetes Dev. Ctries..

[34] Quintas J.B., Mowatt K.B., Mullally J.A., Steinberg A. (2021). New Onset Persistent Hyperglycemia with Initiation of Brentuximab Treatment. Blood.

[35] Visani G., Stefani P.M., Capria S., Malerba L., Galieni P., Gaudio F., Specchia G., Meloni G., Gherlinzoni F., Gonella R. (2014). Bendamustine, etoposide, cytarabine, melphalan, and autologous stem cell rescue produce a 72% 3-year PFS in resistant lymphoma. Blood.

