# Supplementary material for: Combining BeEAM with Brentuximab Vedotin for High-Dose Therapy in CD30 Positive Lymphomas before Autologous Transplantation—A Phase I Study
Source: J Clin Med. 2022 Sep 13;11(18):5378. doi: 10.3390/jcm11185378 (PMC9503567; doi:10.3390/jcm11185378)
Supplement: Supplementary file 1 [file jcm-11-05378-s001.zip › jcm-1835258-supplementary.pdf]

Journal of Clinical Medicine

Supplementary Materials

Supplementary Table S1. Eligibility Criteria.

Inclusion criteria	Exclusion criteria
CD30+ malignant lymphoma in 1st or 2nd remission or 2nd chemo-sensitive relapse	Relevant co-existing disease excluding a treatment according to protocol
HDCT/ASCT planned	Patient not fit for ASCT
Age 18-65	Lack of patient cooperation to allow study treatment as outlined in this protocol.
Written informed consent	Pregnant or lactating female patients.
Negative pregnancy test within 14 days prior to registration (women of childbearing potential)	Concurrent malignant disease (except basalioma/spinalioma of the skin, early-stage cervix carcinoma, or early-stage prostate cancer)
Agreement to use effective contraception from registration until 12 months after completion of treatment	Previous treatment for other malignancies (not listed above) terminated at least 24 months before registration, with no evidence of active disease since then
Platelets $\geq 75x10^9$/L unless there is known marrow involvement of the disease	Major coagulopathy or bleeding disorder.
Absolute neutrophils $\geq 1,5x10^9$/L, unless there is known hematologic/solid tumor marrow involvement	Major surgery less than 30 days before start of treatment.
Total bilirubin < 1.5 x ULN unless the elevation is known to be due to Gilbert syndrome.	Known history of any of the following cardiovascular conditions:<br>- Myocardial infarction in the last 2 years<br>- NYHA Class III or IV heart failure<br>- Current uncontrolled cardiovascular conditions<br>- Recent evidence (6 months before 1st dose of study drug) of LVEF <50%
ALT or AST < 3 x ULN or AST and ALT < 5 x ULN, if the malignancy involves the liver	Symptomatic neurologic disease compromising normal activities of daily living or requiring medications
Serum creatinine < 2.0 mg/dL and/or (calculated) creatinine clearance > 40 mL/min	Any sensory or motor peripheral neuropathy greater than or equal to grade 2
Hemoglobin ≥ 8g/dL.	Patients that have not completed any prior treatment within at least 5 half-lives of last dose of that treatment
	Known hypersensitivity to any excipient contained in the drug formulation of Brentuximab Vedotin
	Acute uncontrolled infection

Supplementary Table S2. Engraftment times of patients undergoing BeEAM compared to BV-BeEAM.

Parameter	BV-BeEAM	Gilli et al.	Hahn et al.	Noesslinger et al.	Redondo et al.	Saleh et al.	Visani et al.
n	12	39	41	41	60	34	43
cHL	7	6	7	9	0	9	15
NHL	5	33	34	32	60	25	28
Age	56 (19-63)	60 (16-71)	NA [1]	52 (22-71)	55 (28-71)	49 (21–68)	47 (18-70)
Prior therapies	2 (1-3)	2 (1-5)	NA	2 (1-4)	NA	2 (1-3)	2 (2-5)
Tc >20 G/L	15 (10-28)	15 (11–46)	12.6 (7–19)	12 (7-110)	NA	NA	13 (8-39)
Tc >50 G/L	26 (16-86)	23 (12–205)	NA	17 (12-NA)	14 (4–53) [2]	13 (7–19)	16 (11-51)
Tc >100 G/L	40 (5-NA)	35 (12–205)	NA	NA	NA	NA	NA
ANC >0.5 G/L	11 (8-17)	11 (9–13)	11.7 (9-15)	NA	11 (9–72) [2]	9 (5–18)	10 (8-12)
ANC >1.0 G/L	12 (10-25)	NA	NA	10 (8-13)	NA	NA	NA

[1] Mean 55, SD 12; [2] excluding one patient (early death).

Supplementary Table S3. Toxicities of BeEAM compared to BV-BeEAM.

Parameter	BV-BeEAM	Gilli et al	Hahn et al.	Noess-linger et al.	Redondo et al.	Saleh et al.	Visani et al.
n	12	39	41	41	60	34	43
cHL	7	6	7	9	0	9	15
NHL	5	33	34	32	60	25	28
Age	56 (19-63)	60 (16-71)	NA [2]	52 (22-71)	55 (28-71)	49 (21–68)	47 (18-70)
Prior therapies	2 (1-3)	2 (1-5)	NA	2 (1-4)	NA	2 (1-3)	2 (2-5)
Duration of hospitalization, d, median (range)	23 (22-56)	27 (20–45)	24.2 (18–33)	25 (21- 86)	26 (15–59)	22 (17–43)	24 (20-57)
**Adverse events**							
Patients with at least one AE (all grades), n (%)	12 (100%)	21 (54%) [3]	100%	NA	NA	NA	NA
Patients with at least one AE (grade III-IV), n (%)	4 (33%)	12 (31%)	NA	NA	NA	NA	NA
Patients with more than one AE (all grades), n (%)	7 (58%)	11 (28%)[3]	NA	NA	NA	NA	NA
Severity of AEs, graded by CTCAE [1], median (range)	1 (1-4)	NA (1-4)	NA	NA	(1-5)	NA	NA
**Adverse events by type, n (%)**							
Cardiac, n (%)	0 (0%)	6 (%)	7 (17%)	NA	5 (8%)	NA	0 (0%)
Dysphagia, n (%)	0 (0%)	7 (18%)	0 (0%)	NA	0 (0%)	NA	0 (0%)
Otorhinolaryngologic, n (%)	0 (0%)	1 (3%)	0 (0%)	NA	0 (0%)	NA	0 (0%)
Endocrine, n (%)	1 (8%)	0 (0%)	0 (0%)	NA	0 (0%)	NA	0 (0%)
Ophthalmologic, n (%)	0 (0%)	3 (8%)	0 (0%)	NA	0 (0%)	NA	0 (0%)
Gastrointestinal, n (%)	1 (8%)	4 (10%)	11 (27%)	NA	60 (100%)	NA	34 (79%)
Infectious, n (%)	12 (100%)	39 (100%)	41 (100%)	NA	46 (77%)	NA	25 (58%)
Metabolism and Nutrition, n (%)	0 (0%)	2 (5%)	0 (0%)	NA	9 (15%)	NA	0 (0%)
Mucosal, n (%)	1 (3%)	27 (69%) [3]	36 (88%)	26 (43%)	59 (98%)	NA	11 (26%)
Musculosceletal, n (%)	0 (0%)	1 (3%)	0 (0%)	NA	0 (0%)	NA	0 (0%)
Nervous system, n (%)	1 (8%)	3 (8%)	0 (0%)	NA	6 (10%)	NA	0 (0%)
Renal and urinary, n (%)	0 (0%)	11 (28%)	13 (32%)	6 (15%)	17 (%)	NA	0 (0%)
Skin, n (%)	0 (0%)	2 (5%)	0 (0%)	NA	1 (2%)	NA	0 (0%)
Vascular, n (%)	0 (0%)	2 (5%)	0 (0%)	NA	1 (2%)	NA	0 (0%)
**Infections**							

Patients with at least one febrile episode (>38.0°C), n (%)	12 (100%)	39 (100%)	41 (100%)	NA	46 (77%)	NA	25 (58%)
Febrile episodes (>38.0°C), median (range)	2 (1-4)	NA	NA	NA	NA	NA	NA
Patients with at least 1 identified pathogen, n (%)	7 (58%)	31 (79%)	23 (56%)	9 (22%)	NA	9 26	3 (7%)
Patients with >1 identified pathogen, n (%)	1 (8%)	16 (41%)	NA	NA	NA	0 (0%)	0 (0%)
Bacteria, Gram-positive, n (%)	5 (42%)	18 (47%)	15 (NA)	NA	NA	6 (18%)	0 (0%)
Bacteria, Gram-negative, n (%)	3 (25%)	12 (30%)	8 (NA)	NA	NA	2 (6%)	0 (0%)
Viral, n (%)	0 (0%)	7 (18%)	6 (NA)	NA	NA	1 (3%)	2 (5%)
Fungal, n (%)	0 (0%)	2 (5%)	1 (NA)	NA	NA	0 (0%)	1 (2%)

[1] Common Toxicity Criteria for Adverse Events; [2] Mean 55, SD 12; [3] not including febrile episodes grade 1.

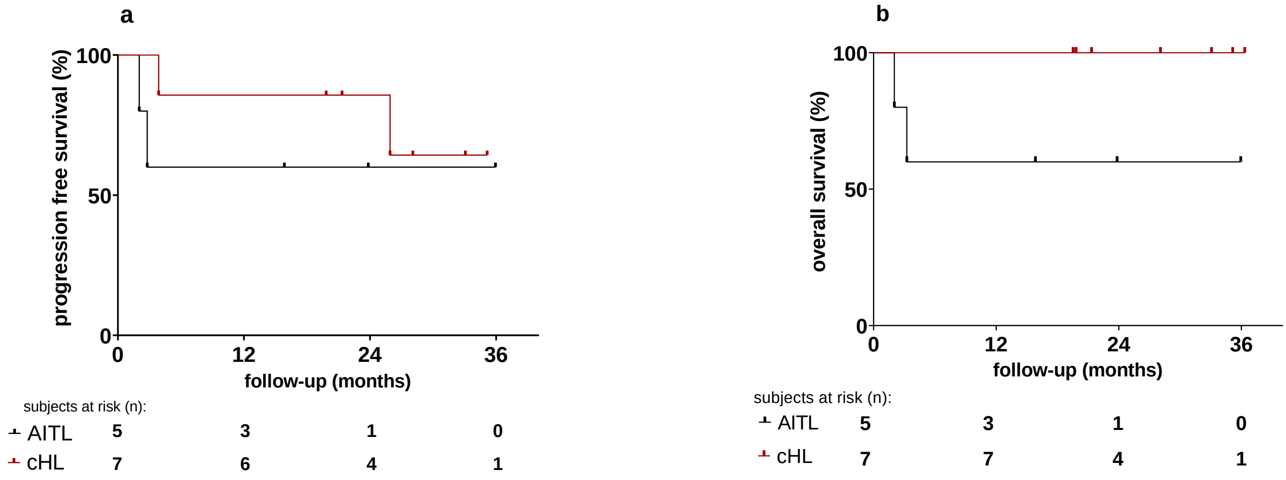

Supplementary Figure S1. Kaplan Maier estimator of cumulative survival rates of 12 patients with CD30 positive lymphoma after HDCT with BV-BeEAM followed by ASCT, stratified by entity: (**a**) progression-free survival; (**b**) overall survival.

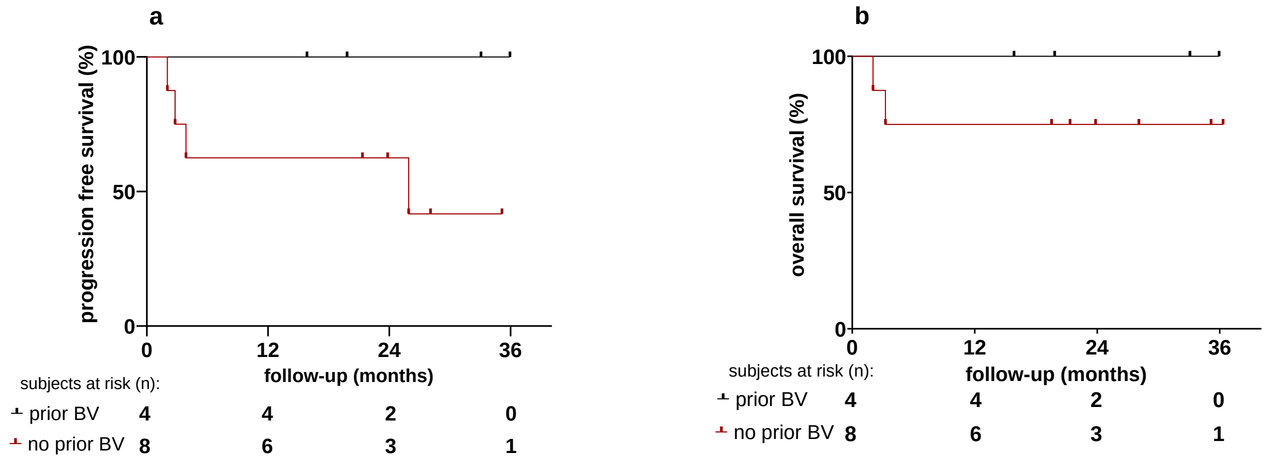

Supplementary Figure S2. Kaplan Maier estimator of cumulative survival rates of 12 patients with CD30 positive lymphoma after HDCT with BV-BeEAM followed by ASCT, stratified by prior exposure to BV: (**a**) progression-free survival; (**b**) overall survival.
